# The Immunological Capacity of Thrombocytes

**DOI:** 10.3390/ijms241612950

**Published:** 2023-08-18

**Authors:** Farzana Ferdous, Thomas Scott

**Affiliations:** 1 Department of Biological Sciences, University of North Carolina at Charlotte, 9201 University City Blvd., Charlotte, NC 28223, USA; 2 Department of Animal & Veterinary Sciences, Clemson University, 129 Poole Agricultural Center, Clemson, SC 29634, USA; trscott@clemson.edu

**Keywords:** thrombocyte, innate immune response, adaptive immune responses, antigen-presenting cell

## Abstract

Thrombocytes are numerous in the blood of aves (birds) and ichthyoids (fish). The origin of this cell type is a common hematopoietic stem cell giving rise to a cell that is active in blood coagulation, inflammatory functions, and the immune response in general. It has been well documented that thrombocytes can phagocytize small particles and bacteria. While phagocytosis with an associated oxidative burst has been reported for chicken thrombocytes, some questions remain as to the degradation capacity of phagosomes in ichthyoids. As innate cells, thrombocytes can be stimulated by bacterial, viral, and fungal pathogens to express altered gene expression. Furthermore, there have been observations that led researchers to state that platelets/thrombocytes are capable of serving as “professional antigen presenting cells” expressing CD40, CD80/86, MHC I, and MHC II. This indeed may be the case or, more likely at this time, provide supporting evidence that these cells aid and assist in the role of professional antigen-presenting cells to initiate adaptive immune responses.

## 1. Overview

The immune system of birds and fish consists of innate and adaptive immunity, which includes cellular and non-cellular (or humoral) components. The cellular components of the non-specific, innate immune response include cells such as macrophages, granulocytes (polymorphonuclear cells such as heterophils), thrombocytes, basophils, eosinophils, and natural killer cells. Since innate cells express various pattern recognition receptors (PRRs), these cells play an important role in the earlier phases of pathogenic invasion. PRRs serve to recognize microbial invasion by detecting pathogen-associated molecular patterns (PAMPs) and harmful products produced by the host body through danger-associated molecular patterns (DAMPs). Upon detection of danger, the cells of the innate immune system respond by producing or releasing molecules such as defensins, cytokines, and chemokines for effector function (i.e., coordinating the recruitment and action of a series of specialized cell populations that fight invading pathogens via phagocytosis and lytic functions). The PRRs on innate cells are also important for initiating the competency of select cells to present antigens to lymphocytes. The lymphocytes (T and B cells) are adaptive immune cells that provide a second layer of protection in a host. Cells such as macrophages and dendritic cells are known to process the pathogenic peptides and present antigens in the context of the major histocompatibility complex (MHC) to lymphocytes to initiate a more specific adaptive immune response to clear infection.

This review focuses on the role of thrombocytes in immune response in birds and fish. Thrombocytes in lower vertebrates such as birds, reptiles, amphibians, and fish are homologous to enucleated platelets in mammals [1]. Nucleated hemocytes found in the hemolymph of invertebrate species perform functions comparable to thrombocytes or platelets [2,3]. In most vertebrate circulation, thrombocytes are the most abundant blood cells after erythrocytes [4,5,6]. Thrombopoiesis occurs in different tissues in adult animals, depending on the vertebrate group and species. In mammals, platelets develop from megakaryocytes [7]. Thrombocytes in birds originate from cells that resemble multipotent hematopoietic progenitors and are produced in the region where the earliest intraembryonic hematopoietic cells develop [8]. Thrombopoiesis occurs in the lymphomyeloid and lymphoid tissues such as the spleen, kidney, and liver in fish [9,10]. Thrombocytes are similar in size to lymphocytes and appear as round, oval, spindle, or spiked cells with long cell processes [1,5,6,11,12,13,14]. The cytoplasm of thrombocytes appears to have a surface-connected canalicular system [15]. Thrombocytes are also capable of producing and releasing a vast array of bioactive proteins that are inflammatory, antimicrobial, and immune-modulating molecules [16,17,18,19,20,21,22,23]. In addition, large, acid phosphatase-positive granules, similar to mammalian lysosomal granules, have been observed in avian thrombocytes [24]. When activated, thrombocytes can release into the circulation numerous intracellular secretory granules (e.g., α granules, dense granules, and lysosomes) that these cells possess.

## 2. Immune Receptors

**Figure 1:**
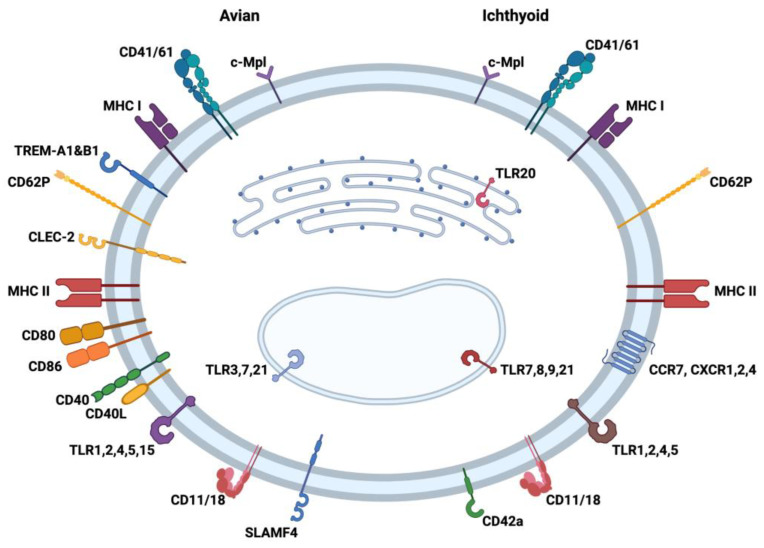
Comparison of immune molecules reported to be present in/on avian (**left**) and ichthyoid (**right**) thrombocytes, which lend support to the unique role of this cell type in innate and adaptive immunity. The figure is divided into left (avian) and right (ichthyoid) sides with several receptors and molecules associated with antigen presentation on the cell surface and in the cytoplasm of thrombocytes. The markers in both bird and fish thrombocytes have been compiled from several refereed publications examining the immune characteristics of this cell type. TLR: toll-like receptor; CD: cluster of differentiation; MHC: major histocompatibility complex; TREM: Triggering Receptors Expressed on Myeloid cells; CLEC: C-type lectin-like receptors; SLAM: signaling lymphocytic activating molecule; CCR: C-C chemokine receptor; CXCR: C-X-C receptor; c-Mpl: thrombopoietin receptor. Created with BioRender.com (accessed on 26 July 2023).

Although platelets and thrombocytes have been known primarily to be involved in thrombosis and hemostasis, these cells have been studied in the last two decades to demonstrate a role in infection, inflammatory functions, and the immune response in general. The detection of PRRs such as toll-like receptors (TLRs) on thrombocytes has led to the discovery of the role of these cells in various innate immune responses. For birds, TLR1, 2, 3, 4, 5, 7, and 21 [25,26] have been identified, and TRL1, 2, 4, 5, 7, 8, 9, 20, and 21 [23,27] are functional for ichthyoid thrombocytes (Figure 1). TLRs 1, 2, 4, and 5 are generally associated with the recognition of patterns associated with bacteria, while TLRs 3 and 7–9 recognize double-stranded and single-stranded RNA often associated with viruses in fish and chicken [28,29]. Subtypes of TLR 1 and 2 are also associated with the recognition of mycoplasma and fungal particles [30]. TLR 20 is detected in fish only and is associated with the recognition of protozoan parasites [31]. TLR 21 is associated with recognizing the CpG dinucleotide sequences in DNA [28,32]. These cells exhibit other PRRs and associated genes such as nucleotide-binding oligomerization domain (NOD)-like receptors (NLR) Family Member X1 (NLRX1), NLR Family CARD Domain Containing 3 and 5 (NLRC3, NLRC5) [33], and C-type lectin receptor (CLR) [34]. The discovery of these molecules associated with pathogen recognition on thrombocytes reinforces the notion that these cells may be playing direct roles in protecting the host from infection like other leukocytes.

In addition to the PRRs, both avian and ichthyoid thrombocytes express markers such as CD41/61 (glycoprotein IIb/IIIa) [35]. This molecule is known to be associated with the activation and aggregation of mammalian platelets [36,37]. The thrombopoietin receptor (c-Mpl) has been identified in birds and fish as a unique marker of thrombocytes related to the proliferation of immature stages of the cell. Thrombocytes additionally express CD62 (P-selectin), which is important cell adhesion molecule for the rolling of platelets and leukocytes on activated endothelial cells [38,39]. The presence of CD11/18 (Complement receptor 3) provides these cells with the ability to attract complement complexes engaged in immune capture by thrombocytes. The chicken thrombocyte exhibits immunoregulatory Ig-like receptors CLEC-2 (C-type lectin) [34], SLAMF4 (CD244, 2B4) [40], and TREM-A1 and -B1 [41,42], whereas CD42a (GPIX) [35] has been observed on the fish thrombocyte. Also, fish thrombocytes have been found to display chemokine receptors (CCR7, CXCR 1 and 2, and CXCR4) [35], while none have yet been identified on the avian cell. The interaction of platelets with neutrophils and other leukocytes is mainly mediated through CD62P and integrins (e.g., CD11b/CD18, CD41/CD61) [43,44]. Since a combination of these adhesion molecules is also observed on thrombocytes (Figure 1), these most likely are important for the interaction between thrombocytes and other immune cells. These interactions emphasize the possible role of thrombocytes as a link between innate and adaptive immunity.

## 3. Immunoregulatory Molecules

**Figure 2:**
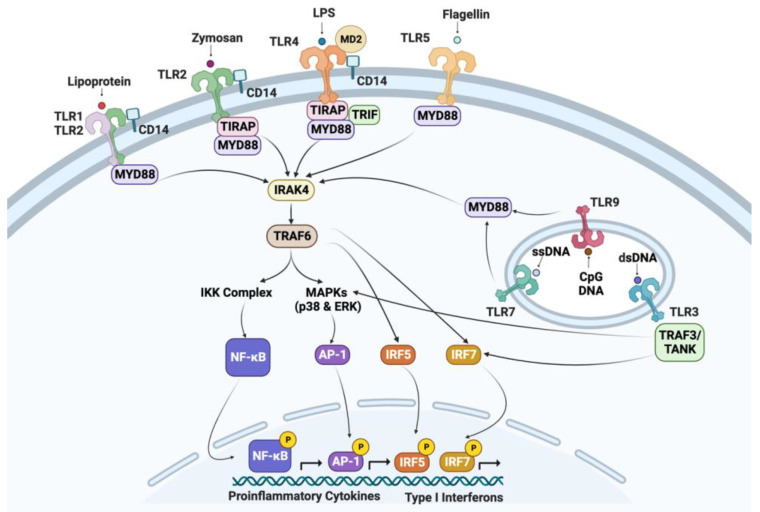
Genes of the TLR signaling pathway observed in chicken thrombocytes. TLR: toll-like receptor; MD2: myeloid differentiation protein 2; CD14: cluster of differentiation 14; LPS: lipopolysaccharide; MyD88: myeloid differentiation primary-response protein 88; TIRAP: Toll/Il-1 receptor domain-containing adaptor protein; TRIF: Toll/Il-1 receptor domain-containing adaptor inducing IFN-β; IRAK: IL-1 receptor-associated protein kinase; TRAF: tumor necrosis factor receptor-associated factor; NF-κB-nuclear factor κB; TANK-TRAF-family-member-associated NF-κB activator; MAPKs: mitogen-activated protein kinases; AP-1: activator protein; IRF: interferon regulatory factor; IKK-IκB kinase; and ERK: extracellular signal-regulated kinase. Created with BioRender.com (Accessed on 26 July 2023).

Thrombocytes have been shown to express, produce, or release a variety of mediators of inflammation, antimicrobial activity, and other immune-modulating activities. PRRs on thrombocytes play an important role in inflammation following microbial infections. Several transcripts associated with signaling downstream of TLRs in chicken thrombocytes were detected in a transcriptome study by our group [33]. In Figure 2, the transcripts detected in chicken thrombocytes were used to compose standard TLR signaling pathways. In response to stimulation of these PRRs, avian thrombocytes are able to produce a variety of bioactive compounds, chemotactic factors (e.g., Macrophage Inflammatory Protein-1 and nitric oxide), and other mediators of inflammation such as inducible nitric oxide synthase (iNOS), cyclooxygenase-2 (COX-2), prostaglandin D2 (PGD2), PGE2, and thromboxane A2 synthases [16,19,45,46]. Chicken thrombocytes express transcripts of anti-inflammatory cytokine transforming growth factor (TGF) and IL-10 [25], and pro-inflammatory cytokines/chemokines (IL-1, IL-6, IL-8, and IL-12) [16,18,19,45]. Chicken thrombocytes have been shown to respond to PAMPs associated with bacterial infection (lipopolysaccharide (LPS) and lipoteichoic acid (LTA)) and viral infection (thymidine homopolymer phosphorothioate oligonucleotide [Poly(dT)] and polyinosinic-polycytidylic acid [Poly(I:C)]) [16,18,47]. Additionally, these cells can induce a pro-inflammatory response to unmethylated CpG oligodeoxynucleotides (ODN) associated with nucleic acids of certain bacteria and viruses [25]. LPS-stimulated chicken thrombocytes have been shown to upregulate transcripts associated with the production of inflammatory cytokines and chemokines, apoptosis, activation of T and B lymphocytes, MAPK activation, IFN activation, and JAK/STAT signaling [33]. Winkler et al. [20] were able to map pathways associated with the response of chicken thrombocytes to LPS stimulation by using inhibitors for kinases such as extracellular-signal-regulated kinase (ERK), p38, mitogen-activated protein kinase kinase (MEK)1/2, and inhibitor of nuclear factor kappa-B kinase (IKK). Chicken thrombocytes have also been shown to have transcripts for platelet-derived growth factors that may have important roles in the vascular system and healing damaged tissue [48,49,50].

Fish thrombocytes also express many genes associated with inflammation including CD62P, IL-1, IL-6, tumor necrosis factor (TNF)α, IFNγ, IL-11, iNOS, TGF, the interleukin receptor common chain, as well as CXC and CC chemokines [21,22,23,51]. The pro-inflammatory cytokines released by leukocytes in response to injury and tissue damage stimulate hepatocytes to produce acute-phase proteins (e.g., C-reactive protein (CRP), serum amyloid A (SAA), metal-binding protein, lysozyme, lectin, etc.) [52]. These proteins are responsible for a variety of defense-related activities such as the inactivation of proteolytic enzymes, preventing the distribution of infectious agents (i.e., either by the destruction of microorganisms or making microbial cells suitable for cell response by modifying surface targets), and the restoration of damaged tissue to a healthy condition. In a study performed by He et al. [22], it was demonstrated that grass carp thrombocytes significantly upregulated the mRNA expressions of some innate immune genes when challenged with bacteria (*Aeromonas hydrophila*) and reovirus (grass carp reovirus). In addition, they determined that the number of thrombocytes in peripheral blood increased after viral and bacterial stimulation, indicating that the increase in the number of thrombocytes in the body may be involved in immune regulation during pathogen invasion.

## 4. Response to Pathogens

Various pathogens that impact the poultry and aquaculture industry have been shown to infect or interact with thrombocytes. According to a study carried out by Schat et al. [53], highly pathogenic (HP) H5N1 avian influenza viruses (AIVs) can infect chicken thrombocytes, which plays a major role in the pathogenesis of this disease. In another study, it has been demonstrated that the AIV strain A (fowl plague virus) is able to replicate in chicken thrombocytes [54]. Another economically important virus, the Newcastle Disease Virus, has also been shown to not only infect chicken thrombocytes but also impair the ability of these cells to perform phagocytosis and migration [55]. On the mammalian side, platelets have been shown to interact with different viruses in different capacities through the various surface receptors for integrins, surface lectins, and TLRs [56,57,58]. Several studies have indicated that during infection with human immunodeficiency virus (HIV), there is a direct interaction of HIV with megakaryocytes and platelets [59]. Viral infections can lead to thrombocytopenia via decreased platelet production or increased platelet destruction [58]. Platelets can also provide protective and pathophysiologic responses during certain viral infections using mediators originating from platelets and the interaction of these cells with other vascular and immune cells [59,60,61,62].

In addition to viruses, parasites have been shown to interact with thrombocytes. Gametocytes of leucocytozoon parasites infect and develop within chicken thrombocytes [63]. In common carp, an infection with the protozoan parasite *Trypanoplasma borreli* leads to severe thrombocytopenia due to nitric-oxide-mediated apoptosis [23]. Nitric-oxide-mediated apoptosis may be the reason for thrombocytopenia in chickens infected with *Plasmodium gallinaceum* [64]. Thrombocytopenia is also observed with infection with most Plasmodium species in humans [65]. However, the mechanisms leading to thrombocytopenia during malaria are not fully understood [65]. Platelets are able to directly interact with and kill circulating plasmodium parasites and infected erythrocytes in patients with malaria. In a study where trout were infected with *Candida albicans*, thrombocytes were observed to interact with erythrocytes, macrophages, other polymorphonuclear cells, and lymphocytes [66]. The thrombocytes appear to form cellular aggregates as rosettes that interact with erythrocytes and macrophages [65]. This phenomenon can also be observed with platelets interacting with erythrocytes, neutrophils, or other cells. Kho et al. [65] have demonstrated that the major parasites associated with human malaria can be killed by platelets.

## 5. Phagocytosis

Phagocytic cells are essential components of the immune system. These cells are responsible for the ingestion and destruction of pathogens, cellular debris, and other foreign elements. PRRs such as TLRs play an essential role in the recognition of pathogens. They trigger both the degradation of pathogens mediated by the release of bioactive, cytotoxic contents from large granules and the subsequent presentation of pathogen-derived peptide antigens. Mammalian platelets are able to bind circulating bacteria and microbial products and present those to neutrophils and other phagocytic cells [67]. In addition to binding and internalizing microorganisms, contact with certain bacteria causes an aggregation and degranulation of platelets. The α-granules of platelets contain and release cationic antibacterial/microbicidal proteins referred to as thrombocidins [68,69] and reactive oxygen species [70]. Thrombocidins support the killing of adherent bacteria [71] and also have been shown to have fungicidal properties in vitro [71].

The phagocytic ability of circulating thrombocytes in chickens was shown first by Glick et al. [72]. Avian thrombocytes are capable of phagocytosing dye particles [24] and several bacterial species including various strains of *Salmonella*, *Escherichia coli*, *Pseudomonas aeruginosa*, and *Burkholderia cepaciao* [24,72,73]. Chicken thrombocytes have been shown to phagocytize about three times as rapidly as heterophils and monocytes, and circulating thrombocytes engulfed nearly twice as many bacteria as the heterophil and monocyte together [4]. The acid-phosphatase-positive granules in avian thrombocytes are considered lysosomal structures associated with phagocytic activity [24].

The phagocytic ability of thrombocytes from other lower vertebrates has also been demonstrated using teleost (*Paralichthys olivaceus*) and amphibian (*Xenopuslaevis*) models [51]. Among peripheral leukocytes in common carp, thrombocytes represent nearly half of the phagocyte population [51]. The process of triggering phagocytosis in carp thrombocytes is closely linked to the presence and activity of factors that are released by LPS from Escherichia coli O55- and phorbol 12-myristate 13-acetate (PMA)-activated leukocytes [74]. Phagocytic particles have been detected in the cytoplasm of thrombocytes of various fish species [35]. In addition, carp thrombocytes exhibit genes for several lysozymes [74] and possess bactericidal activity [75].

Osteichthyoid thrombocytes are considered phagocytes that are fully capable of recognizing opsonized antigens, kill pathogens, and are able to influence the development of both innate and adaptive immune responses [35,51,74]. The detection of phagocytic and antigen-burdened thrombocytes in the spleen and kidneys of common carp indicates a possible role of thrombocytes in the transport of antigens to lymphoid tissues and a contribution to the initiation of adaptive immune response [51]. According to a study carried out by Nagasawa et al. [74], carp thrombocytes efficiently recognized antigens in the presence of inflammatory signals originating from stimulated leukocytes.

The capacity for thrombocytes to express genes encoding various immune factors has been well documented in birds and fish. Several cytokines, chemokines, and immune regulators are produced and secreted to influence other cell types in the immediate vicinity of thrombocyte activation [16,17,20]. These effects range from pro-inflammatory responses and cell migration to an activation/differentiation of effector cells [15,35,76]. The spectrum of gene activation events observed for thrombocytes includes inducible expression as a result of Gram-negative and -positive bacterial ligands to RNA and DNA viral nucleotide sequences [46,47]. The differentiated responses have been reported and result from the engagement of TLRs found on thrombocyte cell surfaces and vesicles [20,25,47]. The soluble factor production of these activation treatments has been shown through bioassays, ELISAs, and Western blotting [16,17,20].

## 6. Participation in Adaptive Immunity

Reports of platelets participating in adaptive immunity number more than a few in the literature [77,78,79,80,81,82]. Ali et al. [78] provided a comprehensive review of the mammalian platelet and its roles in immunity. Discussions of their involvement in adaptive immunity describe platelets as merely assisting APCs through the expression of CD40L (CD154) to engage in the full scope of antigen processing and presentation with the formation of co-stimulatory binding via B7 [83]. Platelets aid DC maturation via CD40L and have the capability to deliver antigens to APCs. More importantly, platelets are able to process and present antigens through MHC I and have been observed to present antigens with MHC II during certain diseases. Class I processing is accomplished in platelets with the cellular mechanism associated with APCs, and the set of co-stimulatory molecules for T-cell activation are present on platelets. Furthermore, platelets appear to enhance T-dependent B-cell responses via CD40:CD40L linkages of these lymphocytes [78,83].

Professional APCs have been well characterized with regard to the properties that define the roles played by dendritic cells (DC), macrophages, and B cells. When implicating a platelet or thrombocyte in APC activity, the comparison will be based upon cellular activity as well as the cell expression of essential surface receptors. Fundamentally, APCs are capable of antigen uptake, express MHC II, provide co-stimulatory activity via B7 (CD80/86), and participate in T-cell activation. All three cell types engulf antigens via endocytosis and/or phagocytosis. With regard to MHC II expression, DCs and B cells constitutively express this molecule, while it is necessary to induce class II on macrophages. B7 molecules are constitutively expressed on DCs but must be induced for expression on both B cells and macrophages. Only DCs and activated B cells stimulate naïve T cells, and effector and memory T cells are activated when the three APC types are expressing MHC II and B7.

**Figure 3:**
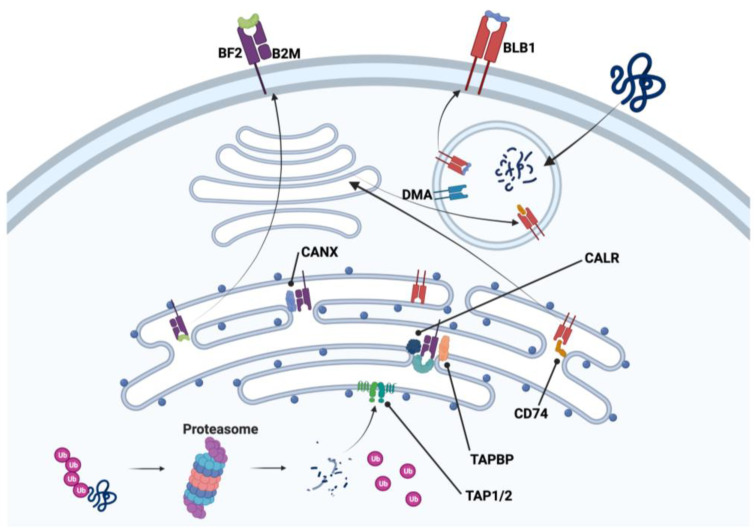
Constitutively expressed genes of class I and II major histocompatibility complex processes of endogenous and exogenous antigen processing pathways found in chicken thrombocytes. B2M: β2-microglobulin; BF2: MCH I alpha chain 2; BLB1: MHC II beta chain; CALR: calrecticulin; CANX: calnexin; CD74: invariant chain of CLIP; DMA: MHC II DM alpha chain; TAPBP: tapasin; TAP1/2: transporter 1/2. Created with BioRender.com (Accessed on 27 July 2023).

Work conducted by our group [33,76] led us to postulate that chicken thrombocytes may indeed support the APC process, if not participate in the induction of adaptive immunity. Our current evidence for this lies in gene expression studies and some initial attempts to observe thrombocyte aggregation at sites of adaptive immune genesis in the spleen [19]. The gene expression profile for the antigen processing and presentation of a chicken thrombocyte is presented in Figure 3. Although not a mammalian platelet, the chicken thrombocyte exhibits immune characteristics, particularly MHC processes, nearly identical to the mammalian platelet [78].

Thrombocytes, like other cells of the body, express MHC I on the cell surface. This imparts individual histocompatibility to the host’s cells, and in the case of thrombocytes, there appears to be cellular connectivity with the MHC I antigen processing mechanisms associated with the endoplasmic reticulum as seen in Figure 3 from our transcriptomic data [33,76]. Table S2 of our publication [33] lists MHC I α-chain (BF2), β2-microglobulin (B2M), and tapasin (TAPBP) as examples of antigen processing and presentation gene expression found in chicken thrombocytes. Transcriptomic results from our other study [66] revealed both MHC I and II expression in chicken thrombocytes that included the MHC I processing/presenting molecules calnexin (CANX), calrecticulin (CALR), and transporter 1 and 2 (TAP1 and 2), and the MHC II processing/presenting molecules MHC II beta chain (BLB2), MHC II DM alpha chain (DMA), and invariant chain of CLIP (CD74). Therefore, foreign proteins can be digested through the proteasome and the resulting antigenic peptides can be bound to the MHC I molecule prior to transport to the cell surface via the Golgi apparatus. Likewise, MHC II produced in the endoplasmic reticulum relocates to the Golgi apparatus and then moves to an endosome to digest the invariant chain of CLIP. This is then followed by CLIP removal and antigen placement in the peptide binding groove with the assistance of DM. This then expands the role of thrombocytes beyond a unique cell to an accessory cell. It not only responds to foreign antigens via a release of soluble factors but also interacts with other immune cells to assist in the orchestration of crucial aspects of immunity [23,25,33,35,51,76].

Equally interesting is the gene expression of MHC II in carp thrombocytes [23,35], lending these cells a potential role in the induction of humoral immune responses like a professional APC. Carp thrombocytes act as phagocytic cells [51,75] and express IL-1β and MHC II [51]. It is apparent from gene expression in chicken thrombocytes that MHC II proteins could be produced and participate in antigen processing per the mechanisms of foreign peptide sequence trafficking in our model (Figure 3). As stated above, Table S2 [33] also provides a list of MHC II gene expressions associated with antigen processing and presentation (e.g., DM). Various roles for chicken thrombocytes in adaptive immunity have been covered by Astill et al. [84], who characterized the avian thrombocyte as a central cell in the immune system. Considering the full range of functions identified for avian and fish thrombocytes, it is apropos to consider this cell as a major component of innate and adaptive immunities in a context greater than blood coagulation and phagocytosis. Indeed, the array of thrombocyte cytokines, chemokines, APC molecules, and co-stimulatory ligands/receptors places it firmly among important immune cells.

The reactivity and subsequent responses to foreign organisms have been mentioned previously in this article. The responses have a typical innate-type component leading to phagocytosis with antigen degradation and the production/release of immune factors (e.g., cytokines and chemokines). The role of these cells in assisting/enhancing DC, macrophages, B cells, and T cells is unquestioned, and their capacity to engage in APC function cannot be denied when several reported studies have revealed strong evidence for antigen processing and presentation. Clearly, the argument for this status is convincing for avian thrombocytes (left side of Figure 1, and Figure 3). Though less documented for the ichthyoid thrombocyte, there is more than sufficient evidence for APC functionality in these non-mammalian species since MHC I and II molecules are present as well as molecules for the associated processing mechanisms. Non-mammalian thrombocytes have MHC I and II molecules on the cell membranes along with co-stimulatory markers CD40, CD40L, CD80, and CD86 on avian thrombocytes. Intracellularly, ichthyoid thrombocytes express LMP and TAP1/2 of MHC I antigen processing [35]. Taking this together with functional evidence for the degradation of foreign proteins strengthens the case for including thrombocytes among other antigen-processing and -presenting cell types.

## 7. Final Thoughts

To fully understand the role of thrombocytes in antigen presentation and interaction with other lymphocytes and APCs, further investigations by many other immunologists are needed. Defining the role of these cells in immune responses will be useful for designing effective vaccines and prophylactics for economically important agriculture species such as poultry and fish. In this new era of mRNA vaccines and our growing knowledge of the cellular activity of the thrombocyte, an improved approach to designing effective vaccines can be undertaken. We are gaining a deeper understanding of the mechanisms employed by thrombocytes as vital participants in both innate and adaptive immunity. In addition to the importance of these cells in blood coagulation, we now have evidence of thrombocyte participation in the activation of sustained immunity for long-term protection.
